# The Mutual Influence of the World Health Organization (WHO) and Twitter Users During COVID-19: Network Agenda-Setting Analysis

**DOI:** 10.2196/34321

**Published:** 2022-04-26

**Authors:** Iman Tahamtan, Devendra Potnis, Ehsan Mohammadi, Vandana Singh, Laura E Miller

**Affiliations:** 1 School of Information Sciences The University of Tennessee Knoxville, TN United States; 2 School of Information Science The University of South Carolina Columbia, SC United States; 3 School of Communication Studies The University of Tennessee Knoxville, TN United States

**Keywords:** COVID-19, agenda setting, network agenda setting, Twitter, social media, public opinion, content analysis, public health, WHO

## Abstract

**Background:**

Little is known about the role of the World Health Organization (WHO) in communicating with the public on social media during a global health emergency. More specifically, there is no study about the relationship between the agendas of the WHO and Twitter users during the COVID-19 pandemic.

**Objective:**

This study utilizes the network agenda-setting model to investigate the mutual relationship between the agenda of the WHO’s official Twitter account and the agenda of 7.5 million of its Twitter followers regarding COVID-19.

**Methods:**

Content analysis was applied to 7090 tweets posted by the WHO on Twitter from January 1, 2020, to July 31, 2020, to identify the topics of tweets. The quadratic assignment procedure (QAP) was used to investigate the relationship between the WHO agenda network and the agenda network of the 6 Twitter user categories, including “health care professionals,” “academics,” “politicians,” “print and electronic media,” “legal professionals,” and the “private sector.” Additionally, 98 Granger causality statistical tests were performed to determine which topic in the WHO agenda had an effect on the corresponding topic in each Twitter user category and vice versa.

**Results:**

Content analysis revealed 7 topics that reflect the WHO agenda related to the COVID-19 pandemic, including “prevention,” “solidarity,” “charity,” “teamwork,” “ill-effect,” “surveillance,” and “credibility.” Results of the QAP showed significant and strong correlations between the WHO agenda network and the agenda network of each Twitter user category. These results provide evidence that WHO had an overall effect on different types of Twitter users on the identified topics. For instance, the Granger causality tests indicated that the WHO tweets influenced politicians and print and electronic media about “surveillance.” The WHO tweets also influenced academics and the private sector about “credibility” and print and electronic media about “ill-effect.” Additionally, Twitter users affected some topics in the WHO. For instance, WHO followers affected “charity” and “prevention” in the WHO.

**Conclusions:**

This paper extends theorizing on agenda setting by providing empirical evidence that agenda-setting effects vary by topic and types of Twitter users. Although prior studies showed that network agenda setting is a “one-way” model, the novel findings of this research confirm a “2-way” or “multiway” effect of agenda setting on social media due to the interactions between the content creators and audiences. The WHO can determine which topics should be promoted on social media during different phases of a pandemic and collaborate with other public health gatekeepers to collectively make them salient in the public.

## Introduction

### Problem Statement

Social media has changed how online users share and receive news and information on various public health emergencies. A contagious and fatal public health emergency that started in early 2020 was the COVID-19 pandemic, with 263,563,622 confirmed cases, including 5,232,562 deaths worldwide as of December 03, 2021 [1]. Right after the outbreak of the virus, social media became the main technology for sharing and receiving information about various aspects of the pandemic.

During the pandemic, social media users received information from various sources such as the news media, politicians, and celebrities. These sources sometimes disseminate biased information due to their politically biased and partisan stance on public issues, consequently impacting public opinion and behavior [2]. Brennen et al [3] found that approximately 20% of misinformation about COVID-19 on social media with a high engagement was posted by gatekeepers, such as politicians, celebrities, and other influential public figures. Thus, even trusted sources of information are likely to produce biased and irrelevant information [3,4], which can develop biased opinions or a false reality about COVID-19.

The widespread distribution of information on social media resulted in the dissemination of low-quality and unverified stories and facts about COVID-19 [3,5,6], increasing public uncertainty about the situation and what will happen next [7].

### Potential Solution

In such situations, trusted sources of public health information such as the World Health Organization (WHO) can develop an agenda to make the public aware of existing challenges and fight against the spread of false information. The WHO agenda can be defined as the topics emphasized and presented to the public at a given time [8].

### Research Questions

To understand and explore the role of the WHO in setting a public health agenda regarding COVID-19 for various social media user categories (ie, health care professionals, academics, politicians, print and electronic media, legal professionals, and the private sector), this study investigated the following research questions:

- Research question 1 (RQ1): “Which topics (ie, agenda) related to the COVID-19 pandemic were promoted by the WHO’s account on Twitter?”
- Research question 2 (RQ2): “What is the relationship between the WHO agenda regarding COVID-19 and the agenda of different categories of WHO followers on Twitter?”
- Research question 3 (RQ3): “How do the WHO and different categories of WHO followers on Twitter affect each other’s agenda regarding COVID-19?”

These research questions help us (1) understand the extent to which the WHO has been successful in setting its agenda about COVID-19 on Twitter and (2) propose how the WHO can more effectively create information-related benefits for different categories of users on Twitter during public health emergencies.

This study adopted the agenda-setting theory [9] as a lens to address the research questions, as it can be used to understand and explain the impact of gatekeepers in shaping public agenda on various issues such as health crises. The importance of agenda setting is that it can be employed by gatekeepers to tell the public what critical issues a country is facing [10] and to impact public opinion about those issues [11]. This study is specifically focused on the network agenda-setting model [12], which states that the salience of interrelationships among attributes (eg, social distancing, handwashing, face covering) of an issue (eg, COVID-19) emphasized by gatekeepers can be transferred to (affect) the public agenda [12].

### Literature Review

#### Agenda Setting

Gatekeepers such as the news media determine which issues are important in society and consequently set the public agenda around those issues [9]. When an issue is being shared frequently and prominently, the public may also come to perceive them as important [13]. For instance, the presentation and repetition of “social distancing” by the WHO on social media could make the majority of people perceive it as an important attribute of COVID-19 that should be given considerable attention.

The agenda-setting theory [9] is about transferring the salience of issues and issue attributes to the public, suggesting that gatekeepers can tell people what to think about and how to think about them [11]. For example, COVID-19 is a public health issue that has a variety of attributes (eg, social distancing, hand hygiene, face covering). The WHO can set an agenda around COVID-19, to bring the importance of COVID-19 and its attributes to the public’s attention.

#### Agenda-Setting Effect

Agenda setting proposes that the repetition of messages about public issues by a gatekeeper can influence people’s minds [13]. As a result, people mentally connect to those issues to a similar degree in which they have been emphasized by the gatekeeper [14]. For instance, by emphasizing social distancing, hand hygiene, and face covering, the public is more likely to make links to these 3 attributes in their minds than other less highlighted attributes.

Agenda-setting research uses correlation tests to investigate how the issue and issue attributes presented by a gatekeeper (representing the gatekeeper agenda) correlate with the issue and issue attributes in the public discourse (representing the public agenda). The assumption is that, if there is a positive correlation between both agendas, the gatekeeper, such as the news media, has been able to impact the public agenda [15].

#### Network Agenda Setting

**Figure 1:**
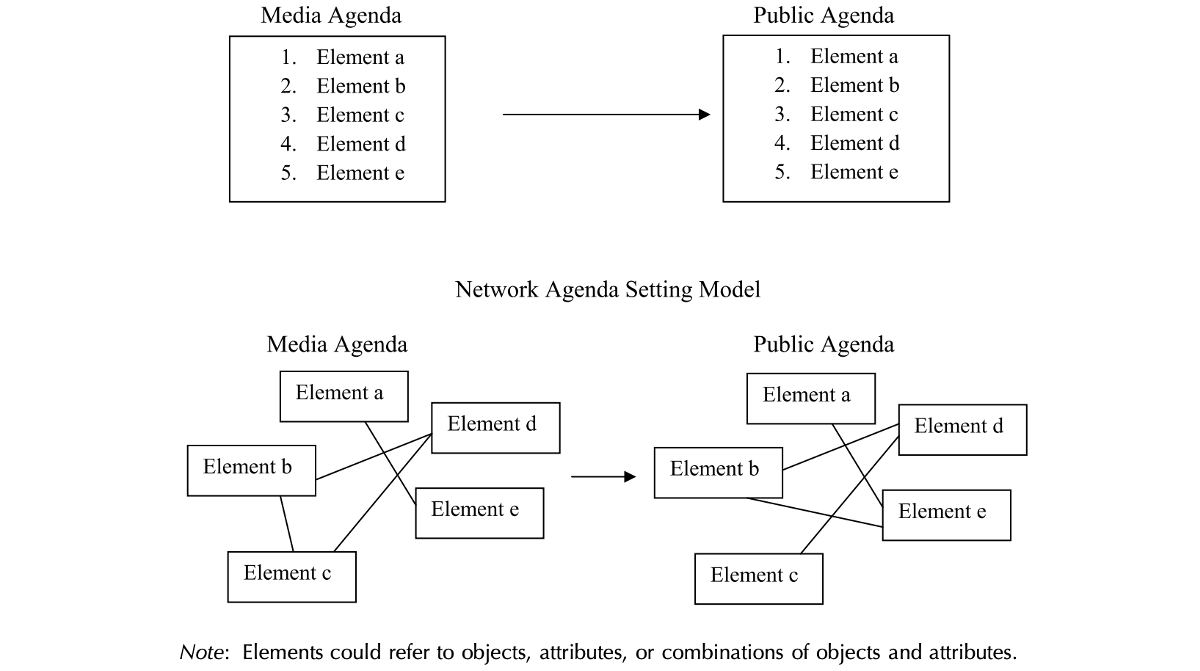
Comparison of a traditional agenda-setting approach with a network agenda-setting model [17].

Guo and McCombs [12] proposed an agenda-setting model, called network agenda setting, according to which gatekeepers like the news media have the power to impact people’s cognitive network. A gatekeeper builds “network connections” between the attributes of an issue and transfers those interconnected attributes to the public’s minds (see Figure 1). For instance, if the news media consider a country’s economic problems to be associated with its foreign policy, audiences are also likely to consider them to be associated with each other [16]. According to network agenda setting, “the more frequently that two elements are associated in the news coverage, the more likely it is that the audience will consider the two interconnected” [16].

Therefore, the attributes of an issue can be transferred to the public as a network of interconnected attributes. For instance, social distancing, handwashing, and face covering, as 3 attributes related to COVID-19, can be transferred to the public agenda simultaneously as a network of attributes [12]. That said, when people think about social distancing, they would also think about handwashing and face covering as measures for protecting against the COVID-19 virus. Therefore, the public can be told not only what issues and attributes to think about and how to think about them but also how to link those issues and attributes in their minds [15].

The main difference between traditional agenda setting and network agenda setting is that the former assumes an issue and its attributes are separately and discretely transferred to the public agenda, while the latter assumes they are transferred simultaneously as a bundle of networked attributes [12]. Figure 1 [18] compares the traditional and network agenda-setting models.

#### Network Agenda-Setting Effect

To identify the network agenda-setting effect, just as with the traditional agenda-setting effect, correlation tests are used to identify how well a gatekeeper’s agenda is correlated with the public agenda. However, in network agenda setting, the agenda will be presented in a network or co-occurrence square matrix (ie, a matrix with the same number of rows and columns) consisting of issue attributes (see Table 1). The value in each cell in the matrix represents how many times the 2 corresponding attributes have co-occurred in a data set: the higher the value, the stronger the 2 attributes are connected. For instance, in Table 1, attributes 3 and 2, with a co-occurrence of 30, are connected stronger than other attributes [18].

Therefore, a matrix represents the agenda network of a gatekeeper or the public for a given issue in a specific period [15]. Assessing the correlation between the 2 matrices can determine if the former has had any agenda-setting effect on the latter [18].

In network agenda-setting studies, social network analysis is applied to illustrate how issue attributes are interrelated [18]. In a network that demonstrates an agenda, each node represents an attribute of an issue, and each tie between any 2 nodes represents their relationships. The number of times the 2 attributes co-occur in a data set represents the strength of the tie [19].

**Table 1:** The matrix of an agenda network for a hypothetical gatekeeper.

Attribute	Attribute 1	Attribute 2	Attribute 3	Attribute 4
Attribute 1	—^a^	15	25	5
Attribute 2	15	—	30	15
Attribute 3	25	30	—	12
Attribute 4	5	15	12	—

^a^Not applicable.

#### Agenda Setting on Social Media

Social media plays an increasingly significant role in agenda setting [20] by making large-scale communication possible and giving voice to different groups of people, such as minorities [21]. The increasing adoption of social media has changed how the news media, politicians, and other influential actors communicate with people and perform their agenda-setting activities [20]. For instance, during elections, candidates and their campaigns make strategic use of social media to mobilize voters by bringing their attention to the issues that are of great public concern [22]. Agenda setting can also be used in health promotion activities on social media [23].

Hemsley [24] stated that the strategic use of social media such as Twitter and its features such as hashtags could establish and promote health, social, political, or environmental agendas. Hashtags and the stories that form around them can become part of people’s social reality and inform their worldviews [24]. For instance, Twitter and hashtags can be used to enhance information dissemination, publicize the movement, invite new people to the movement, enhance its visibility, broadcast messages to broader audiences, and attract people’s attention [25,26].

Lee and Xu [27] also noted that Twitter could be used during elections to set public agendas. For instance, Donald Trump used Twitter and hashtags during the 2016 US presidential election to develop a variety of public agendas, most importantly, the “media bias” and “Clinton’s alleged dishonesty.” Lee and Xu [27] showed that Donald Trump was more successful than Hillary Clinton in drawing public attention to the agendas highlighted by his campaign on Twitter. Feezell [17] indicated that being exposed to political information on Facebook increased perceived issue salience and importance, yielding an agenda-setting effect.

#### The Gap in the Network Agenda-Setting Literature

Past agenda-setting studies have been mostly focused on gatekeepers such as the news media and topics such as political issues (eg, [17,28]). However, it is less studied how global health gatekeepers such as the WHO set an agenda on social media during public health emergencies and what impact they can have on various social media users.

Past studies have suggested that agenda setting can have different effects on various public subgroups [11,19,29]. The network agenda-setting model and the literature in this area does not focus on how different user categories on social media are affected by the gatekeepers they follow on social media.

This study fills in these gaps in the literature, by studying the network agenda-setting effect of the WHO on 6 Twitter user categories (that follow WHO's Twitter account).

## Methods

### Classifying WHO Followers on Twitter to 6 User Categories

The WHO’s followers on Twitter were classified into 6 categories, including (1) health care professionals, (2) academics, (3) politicians, (4) print and electronic media, (5) legal professionals, and (6) the private sector. This classification was done to investigate if and how different categories on social media react to a public health issue.

We adopted and modified the approach used by Toupin et al [30] to classify Twitter users. The main reason for selecting these categories is that they are influential actors in society whose actions can impact citizens during crisis events as explained in the following paragraphs.

In this study, health care professionals include public health workers in academia and industry sectors, such as doctors and nurses. Health care professionals serve citizens during public health emergencies in various ways, for instance, by warning them against self-medicating [31].

Academics were defined as the people who work in academia (except health care professionals), such as professors, researchers, and students who can inform society through their research and writing about various issues related to the COVID-19 pandemic [32].

Politicians include policy and decision makers in the state and federal governments, such as mayors, congressmen, congresswomen, and senators. Politicians can impact people by, for instance, communicating with them about the concerns raised around vaccine safety [33].

Print and electronic media include those responsible for information dissemination and public awareness, such as journalists, press, news agencies, and publishers. The print and electronic media are powerful sources of information for the public as a crisis unfolds [34]. They can, for example, fact-check the information shared on social media related to COVID-19 [35].

Legal professionals include courts, lawyers, and attorneys who provide legal advice and resources, for instance, for an ideal crisis communication strategy [36]. Additionally, they can ensure the compliance of policies with national laws such as human rights [37].

Private sectors include corporations, incorporated organizations, companies, chief executive officers, and for-profits who serve society by satisfying the needs of citizens. For example, companies can take necessary actions to serve their customers by empowering their employees to operate remotely [38].

The classification of followers into 6 categories was done according to the short biographical profiles of Twitter users. The data sets used in this study can be found online [39]. R software was used to collect and analyze the biographies of Twitter users according to the steps in the following paragraphs.

First, the IDs of the approximately 7.5 million Twitter accounts following the WHO in 2020 were retrieved, and the “username” associated with each ID was collected.

Next, the “description” used in biographies of the accounts (ie, usernames) were collected. Table S12 in Multimedia Appendix 1 provides a list of keywords used to classify and identify the Twitter user categories. The list was generated by searching on the Internet and using websites such as the US Bureau of Labor Statistics [40] that provide a list of job titles in various domains. For instance, the keywords used to identify “academics” were as follows: “lecturer,” “professor,” “phd,” “student,” “ph.d.,” “postdoc,” “postdoctoral,” “doctoral,” “msc,” “master,” “ms,” “bs,” “bachelor,” “undergrad,” “grad,” “graduate,” “undergraduate,” “scientist,” “postgrad,” “faculty,” “chancellor,” “university,” “college,” “school,” “provost,” and “vice-provost.”

To make each category as exclusive as possible and increase the reliability of the classification, general keywords, such as “research,” “researching,” “teams,” “organizations,” “institutes,” “campus,” “professional,” “officer,” “change,” “equity,” and “policy,” were excluded in classifying Twitter users. The preliminary investigation and analysis of user biographies indicated that some keywords such as “chief” or “boss” appeared in several categories. Such keywords were also excluded.

### Data Collection

#### Collecting WHO Tweets

Twitter was the main source for data collection. Twitter contains features such as hashtags, retweets, replies, mentions, and likes that make it a suitable platform for studying online social interactions [41]. Using the *brandwach* platform, the current study collected 7090 tweets posted by the WHO from January 1, 2020, to July 31, 2020.

#### Collecting Tweets for the 6 Twitter User Categories

The tweets related to COVID-19 posted by each Twitter user category from January 1, 2020, to July 31, 2020, were collected using the *rtweet* package.

### Data Cleaning

#### Removing Bots

Previous studies have shown that bot accounts are active on Twitter, specifically during political debates, social movements, and public health emergencies. For instance, Ferrara [42] indicated that bots actively promoted political conspiracies during the COVID-19 pandemic. Our study used the *tweetbotornot* package in the R software to classify Twitter accounts into bots and nonbots; the package is 91.78% accurate in identifying bots and 92.61% accurate in identifying nonbots [43]. Accounts that receive a score of at least 50%, or a probability of 0.5, are bots and should be removed from the analysis [44]. Of the 656,805 Twitter accounts in the data set, 441,041 (ie, 67.15%) were classified as bots and consequently excluded from the analysis.

#### Removing Non-English and Non-COVID-19 Tweets

The WHO tweets that included irrelevant keywords such as Ebola, opioids, cancer, tobacco, and malaria were removed. Only the tweets that contained keywords and hashtags related to COVID-19 (see A1 in Multimedia Appendix 1) were included in the study. This approach removed 2111 (ie, 29.77%) of the tweets, leaving 4979 tweets in the WHO data set.

Overall, 7,965,610 tweets written in non-English languages were removed from the data set of WHO followers, leaving 7,547,019 tweets (48.65% of the total tweets) in the data set written in English (see Table S8 in Multimedia Appendix 1). Only the tweets posted by the 6 Twitter user categories that contained at least a keyword or hashtag related to COVID-19 (see A2 in Multimedia Appendix 1) were included in the analysis. This resulted in including 918,976 tweets (ie, 5.9% of the total tweets) related to COVID-19 and written in English in the data set (see Table S8 in Multimedia Appendix 1).

### Content Analysis to Identify the WHO’s Agenda

Content analysis was used to identify the main topics discussed by the WHO in the first half of 2020. These topics constitute the WHO’s agenda related to COVID-19 on Twitter. An agenda is the topics presented in the public or media or any other medium at a given time [8].

The tweets posted by the WHO (n=7090) were stratified by months (January 2020 to July 2020) in Microsoft Excel. Using stratified sampling, 10.00% (n=709) of WHO tweets were randomly selected. The sample size for each stratum (ie, month) was proportional to the number of tweets in the stratum [45], as indicated in Table S9 in Multimedia Appendix 1.

The content analysis was carried out in 3 steps: In the first step, called the training phase, the 2 coders familiarized themselves with the content of the tweets. They coded a set of randomly selected tweets together inductively to increase their comfort level with and learn about the content of tweets. This helped them come to the same understanding of how the tweets should be coded [46]. The second phase, called the pilot test, included coders coding 100 of the tweets from a separate representative sample and assessing the intercoder reliability, independently [47], and resolving the discrepancies. The final step included coding the total sample size, independently, which resulted in an intercoder reliability agreement of 85%.

The coding was done by assigning 1 to 3 keywords that described the tweet's content to each tweet. For example, “handwash” and “handrub” were the 2 keywords assigned to a tweet by the WHO [48].

In analyzing the WHO tweets, the context of the tweets was taken into consideration: The keywords that appeared in more than one topic or those not contextually meaningful were removed. For instance, “support” was removed because it appeared in 3 topics. Each topic was identified by a set of exclusive keywords and hashtags (see Table S11 in Multimedia Appendix 1).

This study ensured the keywords within each topic were representative of that category by several rounds of discussions among researchers. We identified 7 topics in the WHO tweets, including “prevention,” “solidarity,” “charity,” “teamwork,” “ill-effect,” “surveillance,” and “credibility.” These topics constitute the WHO’s agenda about COVID-19 in the first half of 2020. The frequency of the 7 topics in the entire WHO data set was calculated. To identify the relationship between the agenda network of the WHO and its followers, we also identified the frequency of each topic for the 6 Twitter user categories. To do this, the frequency of keywords and hashtags within each topic was calculated and summed.

### Constructing Co-occurrence Matrices and Networks

To create the agenda network for the WHO and each Twitter user category, co-occurrence matrices should be created [12,19,49]. Following the literature (eg, [18,49]), a co-occurrence matrix was created for each of the 7 topics for the WHO and the 6 Twitter user categories. Each matrix included 7 columns and 7 rows. Each row and each column represent a topic related to COVID-19. Each cell contains a digit representing how many times the 2 topics have co-occurred. For example, the cell associated with “teamwork” and “charity” in the WHO matrix has a value of 51, which means that they were mentioned 51 times together by the WHO on Twitter in the first half of 2020. The number of times the 2 topics co-occurred in a data set represents the strength of the tie between those topics [19]. For instance, Table 2 demonstrates the co-occurrence matrix for WHO followers (ie, all 6 Twitter user categories together). To provide a better understanding of what the matrices look like, they were visualized in networks using the *quanteda* library in the R software. The networks associated with the matrices are presented in Multimedia Appendices 2-9.

**Table 2:** The matrix of topics in the data set of World Health Organization (WHO) followers on Twitter.

Topic	Teamwork	Charity	Surveillance	Prevention	Solidarity	Ill-effect	Credibility
Teamwork	0	1303	331	3041	2390	307	208
Charity	1303	0	141	4159	4838	332	86
Surveillance	331	141	0	2849	856	85	82
Prevention	3041	4159	2849	0	13,132	2502	2136
Solidarity	2390	4838	856	13,132	0	1425	682
Ill-effect	307	332	85	2502	1425	0	84
Credibility	208	86	82	2136	682	84	0
Total	7580	10,859	4344	27,819	23,323	4735	3278

### Quadratic Assignment Procedure (QAP)

The quadratic assignment procedure (QAP) was used to assess the correlation between the WHO’s agenda network (ie, matrix) and the agenda network of each Twitter user category. The QAP is a commonly used statistical test in social network analysis and network agenda-setting studies (eg, [49,50]) to calculate the Pearson correlation coefficient between 2 matrices [15]. The QAP indicates whether the correlation between 2 matrices or networks is statistically significant. Once 2 matrices are significantly correlated, the QAP regression test can be used to assess whether an independent variable can predict a dependent variable [18]. QAP linear regression was used to assess if the WHO agenda network could predict the agenda network of each Twitter user category.

### Time Series Modeling (Granger Causality)

#### Overview

Granger causality has been used in previous agenda-setting studies to examine the relationship between media agenda and public agenda (eg, [51-53]). Granger causality was used in the current study to determine if the changes in 1 variable or time series (a series of data points over time) would impact the changes in another time series [54]. According to Granger [54], Y is said to cause X if the current or lagged values of Y can help to predict the future values of X. It determines whether the future value of a dependent variable can be predicted by the past values of an independent variable. Granger causality determines if there is a correlation between the past values of one variable and the present value of another variable [51].

In this study, Granger causality was used, for instance, to examine if “credibility” as a topic that was promoted by the WHO on Twitter predicted the future values of “credibility” in the tweets posted by the WHO’s followers on Twitter. If the *P* value of a Granger causality test is less than .05, the independent variable is said to Granger cause or predict the value of the dependent variable [51]. For instance, it can be said Y Granger caused the values of X. However, it is important to note that Granger causality does not mean causation. In the Results and Discussion sections of this study, “influenced” or similar terms such as “affected” will be used instead of “Granger caused.”

To investigate if each time series in the WHO (eg, teamwork) could predict the value of its corresponding time series (ie, teamwork) in any of the Twitter user categories, a vector autoregression (VAR) model was created. Additionally, to investigate if each time series in any of the Twitter user categories (eg, teamwork) could predict the value of its corresponding time series (ie, teamwork) in the WHO, VAR models were created. Overall, 98 VAR models were created in this study.

To perform Granger causality, first, each variable (ie, topic) should be treated as a time series [53]. This study created time series for each of the 7 topics in the WHO and the 6 Twitter user categories, as explained in the following section.

#### Creating Time Series

To create time series for each topic, the frequency was calculated over time from January 1, 2020, to July 31, 2020, for both the WHO and the 6 categories of WHO followers on Twitter. For each topic, a time series with 182 records was created. Each record contained the frequency of the topic on a specific date. The initial analysis indicated that each time series had many zero number values, which skewed the data. Each zero number represents a lack of data for a given topic on a given date. The highly skewed data violated the normality assumption (see Testing the Residual Normality).

Additionally, having a wide range of values from zero to several hundred led to violating the heteroscedasticity assumption (see Testing the Residual Heteroskedasticity) in many cases. This study tried to resolve the normality and heteroscedasticity issues by aggregating the data to weekly data to reduce zeros. Therefore, for each topic, a time series of 26 weeks was created. Although the data were aggregated to weekly data, the first 2 weeks for most topics still included zero numbers, which again violated the normality assumption in some cases. Removing the first 2 weeks of June 2020 from the analysis resolved the normality issue. Therefore, the analysis was carried out on times series with 24 records (ie, weeks). As an example, the times series for the topics of WHO followers (ie, all 6 Twitter user categories together) are presented in Table S10 in Multimedia Appendix 1.

To perform Granger causality, VAR models should be created first, as explained below.

#### VAR Model

##### Overview

A VAR model is used to determine how 2 or more times series influence each other. In a VAR model, each time series is modeled as a linear combination of past values of itself and the past values of other variables [55]. For instance, a VAR model can be used to determine the relationship between the “teamwork” time series in the WHO (TW) and the “teamwork” time series in one of the Twitter user categories (TU) at time (t). Since there are 2 time series, 2 VAR models should be created: 1 for TW and 1 for TU. The VAR model for TW uses the past values of itself (TW) and the past values of the other variable (TU). In its simple form, the VAR model for TW and TU can be as follows, where TW_t-1_ and TW_t-2_ are the first and second lags of TW (first variable) and TU _t-1_ + TO _t-2_ are the first and second lags of TU (second variable). Each lag is x period ago. For instance, lag one is 1 time period ago or lag two is 2 time periods ago:

TW = TW_t-1_ + TU_t-1_ + TW_t-2_ + TU_t-2_

TU = TU_t-1_ + TW_t-1_ + TU_t-2_ + TW_t-2_

In a VAR model, a different number of lags can be considered for each variable. Thus, the optimal number of lags should be selected using a criterion. This study used the Akaike information criterion (AIC) to select the optimal lag for each VAR model, as explained in the following sections.

##### Lag Selection

The most commonly used criterion in lag selection in VAR models is the AIC [56,57]. The AIC specifies the number of lags to be used in a VAR model [57].

##### Testing the Stationary Nature

A major assumption underlying the VAR model and Granger causality is that time series must be stationary [54]; otherwise, they should be made stationary using the first or higher differences of the variables. A stationary time series has no systematic trend, meaning that its mean and variance do not change over time [58]. Nonstationary time series lead to incorrect inferences [55]. The Augmented Dickey-Fuller Test was used to test whether the variables were stationary [59].

A time series is stationary if the *P* value of the Augmented Dickey-Fuller test is less than .05. In this study, all variables in the WHO and the 6 Twitter user categories were nonstationary, which were transformed to become stationary through differencing (first or higher differences of the variables). Differencing is the process in which the differences between consecutive observations of a variable (ie, time series) are computed [60].

##### Testing the Residual Autocorrelation

A VAR model should be tested to determine if it “provides an adequate description of the data...In time series models, autocorrelation of the residual values is used to determine the goodness of fit of the model. Autocorrelation of the residuals indicates that there is information that has not been accounted for in the model” [61]. The Portmanteau test was used to check the presence of autocorrelation in the models. If the resulting *P* value in this test is less than .05, autocorrelation exists.

##### Testing the Residual Normality

Another assumption underlying the VAR model is that its residuals should be normally distributed; otherwise, inferences may be incorrect. The normality of residuals for all models was tested using the multivariate Jarque-Bera test. After performing this test, when the resulting *P* value is larger than .05, the residuals of VAR model are normal. In 4 cases, BoxCox transformation was used to make the VAR model normal (see Tables S22 and S24 in Multimedia Appendix 1).

##### Testing the Residual Heteroscedasticity

Another assumption underlying the VAR model is that there should be no heteroscedasticity in residuals. Heteroscedasticity refers to a condition in which the variance of the residual in a regression model varies widely. To test heteroscedasticity, the Autoregressive Conditional Heteroscedasticity-Lagrange Multiplier (ARCH-LM) test proposed by Engle [62] was used. Once the test is performed, if the resulting *P* value is larger than .05, there is no heteroscedasticity in the data. No heteroscedasticity was observed in the VAR models.

## Results

### Results of Content Analysis

The first study objective was to identify the topics discussed by the WHO on Twitter about COVID-19. Using content analysis, 7 topics were identified inductively, including prevention (n=2430), solidarity (n=717), teamwork (n=601), surveillance (n=276), charity (n=243), ill-effect (n=213), and credibility (n=156). The numbers in the parentheses represent the frequency of each topic.

“Prevention” refers to the tweets posted by the WHO about how to avoid contracting the virus, including content about disinfecting surfaces, hand washing, wearing masks, vaccination, social distancing, isolation, and staying home. “Solidarity” refers to the tweets emphasizing the importance of unity, resilience, kindness, or supporting groups like refugees. “Teamwork” was another topic discussed by the WHO to highlight that countries, governments, organizations, and people should collaborate, coordinate, cooperate, be committed, and be accountable to control the pandemic. “Surveillance” emphasizes that governments should keep tracing, investigating, monitoring, and screening people and regions affected by the virus to take necessary actions. “Charity” refers to the donations, fundraisings, and financial support received by the WHO from governments, organizations, companies, celebrities, or other countries to fight against the virus. “Ill-effect” refers to the WHO's tweets about the consequences of the virus, such as disruptions in the economy, trades, health systems, mental health, abuse, and home violence during the quarantine. “Credibility” includes the tweets that demonstrate the importance of facts and bring people’s attention to the rumors, misinformation, and fake information related to COVID-19.

This study also calculated the frequency of these topics for the 6 Twitter user categories and all of them together (hereafter, WHO followers). In the data set of WHO followers, prevention (228,700) had the highest frequency, followed by solidarity (72,192), charity (28,870), teamwork (23,879), ill-effect (15,151), surveillance (13,761), and credibility (11,726). Table S13 in Multimedia Appendix 1 presents the frequency of these topics in the WHO data set, WHO followers data set, and data set of each Twitter user category.

There was a strong tie between “prevention” and “solidarity” in in the matrix of the WHO and WHO followers and all 6 Twitter user categories. The strong tie between “solidarity” and “prevention” could indicate that the former is vital for the prevention of COVID-19 (or even treatment and response to the virus). For instance, to help prevent the spread of the virus, people should be united in following public health guidelines such as staying home, hand washing, and social distancing. These connections could represent social realities constructed around COVID-19 on Twitter and be transferred to the public agenda through network agenda setting [53].

### Results of the QAP and QAP Regression

The QAP was used to measure the similarity between agenda matrices (networks). The QAP calculates the Pearson’s correlation coefficient between the 2 matrices. The co-occurrence matrices for the WHO followers and WHO are presented in Table 2 and Table 3, respectively. Other matrices are presented in Tables S14 to S19 in Multimedia Appendix 1.

Degree centrality is an important concept in network analysis, which indicates how important a node within the network is and will be calculated by the total number of connections a node has [63]. In this study, nodes are topics. The most central topic on the WHO agenda network is “prevention,” with 483 degrees of centrality, followed by “solidarity,” with 420 degrees of centrality (see the row that shows the Total in Table 3). The most central topic on the WHO followers’ agenda is “prevention,” followed by “solidarity” and “charity” (see the row that shows the Total in Table 2). The 2 most frequently linked topics on the WHO agenda network and WHO followers’ agenda network are “solidarity” and “prevention” because they have co-occurred 150 times.

Results of the QAP tests indicated a positive and high correlation between the WHO’s agenda network and the agenda network of WHO followers (see Table 4). The QAP correlation tests also showed that the WHO agenda network and the agenda network of Twitter user categories are significantly correlated. According to network agenda setting [12], these results provide evidence that the salience of interrelationships among topics can be transferred from the WHO to the agenda of its followers on Twitter.

QAP linear regression was also carried out to assess whether the WHO agenda network (the independent variable) could predict the agenda networks of Twitter user categories (the dependent variables). As evident in Table 5, the WHO agenda network could predict all dependent variables, providing evidence that the agenda network of the WHO can impact the agenda network of Twitter user categories. For instance, the adjusted R-squared for politicians is 0.71, indicating that the WHO can explain 71% of the variance in the politicians’ agenda network. The WHO also explains 62% of the variance in the network of WHO followers.

**Table 3:** The matrix of topics in the World Health Organization (WHO) data set on Twitter.

Topic	Teamwork	Charity	Surveillance	Prevention	Solidarity	Ill-effect	Credibility
Teamwork	0	51	23	110	118	9	6
Charity	51	0	1	37	90	3	2
Surveillance	23	1	0	101	14	2	0
Prevention	110	37	101	0	150	48	37
Solidarity	118	90	14	150	0	28	20
Ill-effect	9	3	2	48	28	0	3
Credibility	6	2	0	37	20	3	0
Total	317	184	141	483	420	93	68

**Table 4:** The quadratic assignment procedure (QAP) correlations between the World Health Organization (WHO) agenda network and agenda network of WHO followers and Twitter user categories.

Twitter user categories	Correlation (*r*) with the WHO agenda matrix	*P* value
Politicians	0.85	.001
Private sector	0.79	.01
Print and electronic media	0.77	.01
Legal professionals	0.80	.01
Health care professionals	0.79	.001
Academics	0.79	.01
WHO followers	0.80	.01

**Table 5:** The quadratic assignment procedure (QAP) linear regression for the World Health Organization (WHO) agenda network (independent variable) and networks of Twitter user categories (dependent variables).

Dependent variables	*F* statistic	Coefficient	Adjusted R-squared	*P* value (2-tailed)
Politicians	104.90	5.80	0.71	.002
Private sector	66.22	4.90	0.61	.005
Print and electronic media	60.43	9.50	0.59	.008
Legal professionals	71.95	3.20	0.63	.005
Health care professionals	61.70	13.60	0.60	.001
Academics	67.89	17.00	0.62	.004
WHO followers	70.01	51.20	0.62	.001

### Results of Granger Causality

By performing 98 Granger causality tests, this study examined if the 7 topics in the WHO agenda Granger caused or predicted the future values of the topics in the tweets by the 6 types of Twitter users or vice versa. For instance, we tested to see if “teamwork” in the WHO Granger caused (ie, predicted the future values of) “teamwork” in health care professionals. We also investigated if “teamwork” in health care professionals Granger caused “teamwork” in the WHO. This study uses the term “influenced” or “affected” instead of “Granger caused” or “predicted the value of.”

Tables 6 and 7 provide summaries of the Granger causality tests for the WHO and WHO followers. The summary of the Granger causality tests for other Twitter user categories is presented in Table S20 to Table S25 in Multimedia Appendix 1. Among the 7 topics, only “surveillance” in the WHO influenced “surveillance” in WHO followers (*F*_5,10_=4.74, *P*=.01). On the other hand, among the 7 topics in the WHO followers, “charity” influenced “charity” in the WHO (*F*_6,6_=7.48, *P*=.01), and “prevention” influenced “prevention” in WHO (*F*_5,10_=4.69, *P*=.01).

The results indicated that the WHO influenced “surveillance” in politicians (*F*_6,6_=5.13, *P*=.03) and “surveillance” in print and electronic media (*F*_5,10_=9.33, *P*=.001). Additionally, the WHO influenced “ill-effect” in print and electronic media (*F*_5,10_=4.02, *P*=.02), “credibility” in the private sector (*F*_5,10_=7.12, *P*=.001), and “credibility” in academics (*F*_5,10_=12.5, *P*=.001).

Twitter user categories also influenced several topics in the WHO’s agenda: “credibility” in academics (*F*_5,10_=6.10, *P*=.001), “credibility” in politicians (*F*_5,8_=8.06, *P*=.001), and “credibility” in the private sector influenced “credibility” in the WHO (*F*_5,10_=4.33, *P*=.02). “Charity” in health care professionals (*F*_6,6_=37.93, *P*=.001) and “charity” in academics influenced “charity” in the WHO (*F*_6,6_=4.62, *P*=.04). “Prevention” in politicians (*F*_5,10_=13.21, *P*=.001) and “prevention” in print and electronic media (*F*_5,10_=15.04, *P*=.001) influenced “prevention” in the WHO. Print and electronic media influenced “surveillance” (*F*_5,10_=3.7, *P*=.04) in the WHO. The private sector also influenced “ill-effect” in the WHO (*F*_5,10_=7.35, *P*=.001). The WHO and legal professionals did not influence each other in any of the 7 topics.

**Table 6:** Granger causality test for World Health Organization (WHO) topics as independent variables and the topics of WHO followers as dependent variables.

Topics	*F* test	*P* value
Teamwork	0.04	.99
Charity	0.31	.91
Surveillance	4.74	.01
Prevention	0.27	.92
Solidarity	0.16	.97
Ill-effect	1.49	.29
Credibility	2.57	.11

**Table 7:** Granger causality test for the topics of World Health Organization (WHO) followers as independent variables and WHO topics as dependent variables.

Topics	*F* test	*P* value
Teamwork	0.49	.78
Charity	7.48	.01
Surveillance	1.70	.22
Prevention	4.69	.01
Solidarity	0.93	.50
Ill-effect	0.66	.66
Credibility	1.23	.38

## Discussion

### Contribution to Agenda Setting

#### Who Sets the Agenda?

Rarely any previous network agenda-setting study has investigated the relationship between a gatekeeper’s agenda network and the agenda networks of various types of social media users, specifically in the context of public health emergencies.

This study found that, although there was a high correlation between the WHO agenda network and agenda network of Twitter user categories, the WHO influenced only some topics related to COVID-19 in the 6 Twitter user categories and vice versa (see the Granger causality test results). For instance, the WHO only influenced “ill-effect” in print and electronic media. Likewise, different Twitter user categories only influenced some (not all) topics in the WHO agenda.

It is hard to say who is leading the overall trends and topics related to COVID-19 on Twitter, mainly because social media provides unlimited space where various sources can interact with and impact each other [15]. However, it seems that there is less interaction between the WHO and some Twitter user categories during public health emergencies. For instance, neither legal professionals nor the WHO influenced each other. It is possible that legal professionals are not influenced by the WHO because they naturally often perform independently and remain impartial of external resources.

This study was not designed to measure the impact of the WHO on top influencers within each type of Twitter user or vice versa. Future research can explore who sets the agenda and who establishes the agenda-setting effect first. It is possible that the WHO originates the tweets about COVID-19, but the top influencers within each Twitter user category set the agenda or promote the agenda already set by the WHO, through retweeting the WHO’s tweets.

#### Two-Way Agenda-Setting Effect

This study informs the network agenda-setting model by demonstrating that there can be a “2-way” relationship between the agenda of the WHO and its followers on Twitter. Vargo and Guo [53] also stated that the media agenda is reciprocal, in that network agenda setting is more complex than what past traditional agenda-setting studies have suggested. Evaluating the network agenda-setting effects on social media is more complex than other platforms because, for instance, the agenda of WHO followers about COVID-19 could have been influenced by other resources too, such as the Centers for Disease Control and Prevention (CDC), providing initial evidence for a “multidirectional” network agenda setting effect.

Thus, the WHO does not seem to set the public agenda in a unidirectional nature. Neither the WHO nor Twitter user categories play a leading role on social media because they influence each other’s agenda. It is possible that the WHO and different types of Twitter users pay attention to each other’s agenda and interact with each other on some topics related to COVID-19 in a bidirectional way (or multidirectional way).

Vargo and Guo [53] also suggested that different news media pay attention to and are impacted by each other’s agenda. Therefore, agenda setting is not always a 1-way communication mechanism from the mainstream media to the public. Actually, by making large-scale communication possible through social media, agenda setting is no longer only in the control of certain types of users [21]. An agenda can be created by laypeople on social media and shape the media agenda or vice versa [64].

### Two Levels of the Agenda-Setting Effect on Twitter

A limitation in most network agenda-setting studies is that it is not clear if the public agenda is directly impacted by gatekeepers (eg, news media) or if other sources are involved too in influencing public agenda. For instance, the study by Vu et al [15] compared the most prominent issues in the national news media in the United States from 2007 to 2011 (extracted from the Pew Research Center’s PEJ) with public opinion extracted from the Gallup Poll results (which has been surveying the public since 1939 about the most important problems facing the United States). In such studies, it is hard to determine if the national news media impact public agenda or whether other sources are also involved. One way to minimize this methodological limitation is to analyze the opinions of the users who follow a gatekeeper’s account on social media or to analyze those users subscribed to a news media channel, such as CNN.

To fill this gap, this study investigated 6 Twitter user categories that follow the WHO Twitter account. From the results of this study, it can be concluded that there are 2 levels of the agenda-setting effect on Twitter—one at the aggregate level (see [19]), that is all social media users (eg, WHO followers on Twitter), and another at the Twitter user category level, in which each user category would be influenced differently by the agenda that is set by a gatekeeper. It may also be the case that the interaction among the agenda networks of all Twitter user categories builds the overall Twitter agenda network.

### Practical Implications for the WHO

The results of this study can inform policy and be used to prepare for future pandemics in several ways: First, the WHO should define a clear strategy as how to use social media during pandemics to convey its messages to the public. The WHO should have a plan for what topics are more critical for the public during similar public health emergencies and work on transferring them to the public agenda.

Second, how messages are framed and presented to the public is important. The WHO can identify the needs of social media users and provide information-related benefits for them by framing and presenting more effective messages during public health emergencies. The strategic framing of public health messages can help the WHO to have greater impact on social media users. For instance, to design more effective messages and attract more audiences, the WHO can frame its messages by using hashtags that are popular among the public [65].

Third, using hashtags can lead to establishing and promoting important public health, social, political, or environmental agendas [24]. Hashtags have various functions, such as information searching and discovering, information organization, information distribution, information collection, and protecting information [26]. Our analysis of WHO tweets indicated that the WHO had used only a few hashtags related to COVID-19 in its tweets, including #safehands, #togetherathome, #handhygiene, #unitedagainstcoronavirus, and #stayhome. The WHO can use more hashtags during public health emergencies to convey its messages to people and create more engagement with them [65]. For instance, WHO could frame its message about “credibility” of information, using a hashtag like “#WHOFactChecker, as follows: “#WHOFactChecker recommends that you continue to take appropriate actions to protect yourself and those around you in the summer as there is no evidence that warm weather can kill the #COVID-19 virus.”

Fourth, this study suggests that there could be a “2-way” or “multiway” agenda-setting effect on social media. For instance, the WHO, the CDC, the US government, and politicians could all interact with and influence each other’s agenda. Therefore, in future public health emergencies, the WHO can determine which topics should be promoted on social media during different phases of a pandemic and collaborate with other gatekeepers such as the CDC to collectively make them salient in the public.

### Limitations and Future Research

This study investigated only 6 Twitter user categories. There are other types of users following the WHO on Twitter, which can be studied in future work, such as artists and athletes.

Another limitation of using Twitter data is that Twitter users are not representative of the entire population.

The agenda of WHO followers about COVID-19 could have been influenced by other resources too, such as the CDC or any other government agency. Future studies should find ways to also take the effect sources other than the WHO into consideration. Surveys and interviews with WHO followers on Twitter can provide more insights into the WHO's impact on social media users’ opinions about COVID-19.

The 7 topics promoted by the WHO on Twitter could possibly also be found in the messages shared on Twitter by other resources such as the US government. It could be the case that the 6 categories of WHO followers on Twitter were also propagating the agenda by other resources and not specifically that of the WHO. Therefore, although this study suggests that there can be a “2-way” agenda-setting effect on social media, there could be a “multiway agenda-setting effect” on social media.

This study was limited to tweets written in English. Analyzing tweets in other languages may provide new insights on the agenda-setting effect of the WHO on Twitter.

Although this study analyzed a sample of tweets in the first 6 months in 2020, it is possible that the analysis of all the tweets posted by the WHO and its followers in the second half of 2020 could lead to different findings.

We attempted to make the Twitter user categories as exclusive as possible; however, there might be some overlap among different categories. For instance, a Twitter user account could fall into the academics and print and electronic media categories.

Future studies in this area can also determine to which social media user categories and top influencers within those categories the WHO should reach out if it wants to have more impact on social media users.

Future studies in this area can also study whether and to what extent the WHO has made strategic use of hashtags to communicate its messages about COVID-19 to the public. Future research can also investigate the role of hashtags used by the WHO in setting its COVID-19 agenda and the effect of that agenda on public opinion.

Although using social media data provides a legitimate method for studying the agenda-setting effect of WHO, more empirical research, including surveys and interviews, can be used in future studies to understand the role of the WHO in shaping public agenda on social media.

The results show that the WHO and Twitter user categories of different types could influence some of the COVID-19–related topics in each other; however, this study did not investigate whether different Twitter user categories would also impact each other’s agenda network. This impact can be examined in future studies by exploring, for instance, the network agenda-setting effect between academics and health care professionals on Twitter.

### Conclusions

This is among the first studies that demonstrate the presence of network agenda-setting effects between the WHO and its followers on Twitter, specifically different Twitter user categories. In line with the network agenda-setting model, this study showed that the topics promoted by the WHO about COVID-19, such as “credibility” or “surveillance,” were linked together in a network.

This study extends theorizing on agenda setting by providing evidence that agenda–setting effects vary by different Twitter user categories and topics. For instance, the WHO only influenced “surveillance” in politics and print and electronic media, or health care professionals only influenced “charity” in the WHO, while the WHO and legal professionals did influence each other.

This study also extends theorizing on agenda setting by indicating that, while network agenda setting is known as a “1-way” model, there can be “2-way” or “multiway” effects of agenda setting on social media, because the influences between the WHO and Twitter user categories were reciprocal on Twitter.

## Appendix

Multimedia Appendix 1 Supplementary files.

Multimedia Appendix 2 Agenda network of the World Health Organization (WHO).

Multimedia Appendix 3 The agenda network of WHO followers (six categories together).

Multimedia Appendix 4 The agenda network of politicians.

Multimedia Appendix 5 The agenda network of the private sector.

Multimedia Appendix 6 The agenda network of print and electronic media.

Multimedia Appendix 7 The agenda network of legal professionals.

Multimedia Appendix 8 The agenda network of health care professionals.

Multimedia Appendix 9 The agenda network of academics.

## Abbreviations

AIC: Akaike information criterion
ARCH-LM: Autoregressive Conditional Heteroscedasticity-Lagrange Multiplier
CDC: Centers for Disease Control and Prevention
QAP: quadratic assignment procedure
RQ: research question
VAR: vector autoregression
WHO: World Health Organization
