# Supplementary material for: The Mutual Influence of the World Health Organization (WHO) and Twitter Users During COVID-19: Network Agenda-Setting Analysis
Source: J Med Internet Res. 2022 Apr 26;24(4):e34321. doi: 10.2196/34321 (PMC9045487; doi:10.2196/34321)
Supplement: Multimedia Appendix 1 [file jmir_v24i4e34321_app1.docx]

# Multimedia Appendix

Table S8. Twitter user categories and their COVID-19 related tweets in English from January 1 to July 31, 2020.

|  | Twitter user accounts | Frequency of Bot accounts removed | Frequency of tweets by non bot accounts | Frequency of tweets written in English | Frequency of tweets written in English related to COVID-19 |
| --- | --- | --- | --- | --- | --- |
| Academics | 236,494 | 17,0443 | 3,752,817 | 2,513,946 | 296,431 |
| Politicians | 39,259 | 24,358 | 804,296 | 595,710 | 66,132 |
| Print and electronic media | 162,934 | 96,804 | 2,720,268 | 2,082,200 | 197,522 |
| Healthcare professionals | 125,572 | 93,380 | 6,953,100 | 1,404,590 | 237,166 |
| Legal professionals | 39,244 | 24,745 | 545,844 | 380,043 | 54,493 |
| Private sector | 53,302 | 31,311 | 736,304 | 570,530 | 67,232 |
| All | 656,805 | 44,1041 | 15,512,629 | 7,547,019 | 918,976 |

Table S9. Sample size of WHO tweets.

| The months when the tweets were posted by WHO | Frequency of tweets | Proportion of tweets in the final sample (10% of the population) |
| --- | --- | --- |
| January 2020 | 816 | 82 |
| February 2020 | 1,350 | 135 |
| March 2020 | 1,287 | 129 |
| April 2020 | 1,402 | 140 |
| May 2020 | 1320 | 132 |
| June 2020 | 915 | 92 |
| Total | 7,090 | 709 |

Table S10. Time series for the seven issues of WHO followers.

| Week | Teamwork | Charity | Surveillance | Prevention | Solidarity | Ill-effect | Credibility |
| --- | --- | --- | --- | --- | --- | --- | --- |
| 3 | 4 | 0 | 25 | 78 | 4 | 1 | 1 |
| 4 | 87 | 23 | 142 | 1043 | 111 | 6 | 51 |
| 5 | 99 | 33 | 154 | 1360 | 266 | 24 | 213 |
| 6 | 54 | 41 | 56 | 696 | 187 | 14 | 83 |
| 7 | 115 | 34 | 55 | 657 | 191 | 22 | 80 |
| 8 | 170 | 42 | 90 | 676 | 118 | 44 | 91 |
| 9 | 316 | 157 | 294 | 4625 | 650 | 91 | 293 |
| 10 | 482 | 226 | 376 | 7448 | 1256 | 128 | 450 |
| 11 | 1961 | 1262 | 1171 | 22419 | 6107 | 717 | 1248 |
| 12 | 2065 | 3427 | 1162 | 30776 | 10397 | 1054 | 1311 |
| 13 | 2535 | 3892 | 1075 | 26190 | 9351 | 1407 | 1862 |
| 14 | 2272 | 2833 | 1004 | 19813 | 7282 | 1531 | 1004 |
| 15 | 1927 | 2315 | 930 | 14484 | 5759 | 1051 | 612 |
| 16 | 1802 | 3551 | 936 | 13808 | 5185 | 1122 | 736 |
| 17 | 1486 | 2067 | 895 | 12095 | 4175 | 977 | 473 |
| 18 | 1211 | 1901 | 787 | 11312 | 3697 | 857 | 728 |
| 19 | 1202 | 1169 | 770 | 8492 | 2970 | 852 | 337 |
| 20 | 1208 | 1259 | 680 | 9257 | 2872 | 919 | 350 |
| 21 | 922 | 942 | 610 | 7596 | 2308 | 716 | 358 |
| 22 | 972 | 812 | 631 | 7059 | 2099 | 910 | 316 |
| 23 | 756 | 776 | 495 | 6830 | 1823 | 680 | 309 |
| 24 | 770 | 727 | 514 | 7057 | 1813 | 837 | 282 |
| 25 | 742 | 732 | 480 | 7295 | 2063 | 718 | 281 |
| 26 | 721 | 656 | 421 | 7672 | 1,523 | 476 | 266 |

Table S11. The keywords representing each of the seven issues promoted by WHO on Twitter.

| Issues | Frequency | Description | Keywords and hashtags |
| --- | --- | --- | --- |
| Prevention | 2430 | Disinfecting surfaces, hand washing, wearing masks, vaccination, social distancing, isolation, and staying home. | cleanser, handrub, clean, cover, cleaning, disinfect, handwashing, preventive, precautions, mask, masks, handwash, hygiene, preventing, wash, prevent, washing, preventable, prevention, sanitation, wear, covering, #safehands, #handhygiene, handwashing, vaccines, vaccine, vaccinated, vaccination, vaccinations, vaccinated, #vaccineswork, #vaccines, protect, protecting, protective, protects, lockdown, quarantine, lockdowns, isolate, isolation, isolated, #halthyatHome, #togetheratHome, #beactive, #stayhome, #stayathome, distance, distancing |
| Solidarity | 717 | Unity, resilience, kindness, supporting groups like refugees. | #unitedagainstcoronavirus, #withrefugees, compassion, help, humanitarian, kindness, resilience, solidarity, unity |
| Teamwork | 601 | Collaboration, coordination, cooperation, being committed, and being accountable. | Contributing, coordinate, accountability, cooperation, contributions, collaboration, collective, commitment, commitments, coordinating, contribution, leadership, preparedness, resolution |
| Surveillance | 276 | Tracing, investigating, monitoring, and screening people and regions affected by the virus to take necessary actions. | tracing, investigation, trace, monitor, monitoring, screening, surveillance |
| Charity | 243 | Donations, fundraisings, and financial support received by WHO from governments, organizations, companies, and celebrities. | donation, pledged, donations, fundraisers, pledges, financial, fund, donate, donated, pledge, fundraiser, donating, funders, funding, pledging |
| Ill-effect | 213 | Consequences of the virus such as disruptions in the economy, trades, health systems, mental health, abuse, and home violence during the quarantine. | disruptions, consequences, disrupted, disruption, depression, #endviolence, #mentalhealth, #stopelderabuse, abuse, threatens, violence |
| Credibility | 156 | Tweets about facts, rumors, misinformation, and fake information related to COVID-19 | facts, rumors, misinformation, fact, #knowthefact, #knowthefacts, #takecarebeforeyoushare |

Table S12. The keywords used to classify WHO followers to six Twitter user categories.

| Twitter user categories | Keywords used to analyze the descriptions used in the Twitter users accounts |
| --- | --- |
| Academics (except healthcare professionals) | lecturer, professor, PhD, student, Ph.D., postdoc, postdoctoral, doctoral, msc, master, MS, BS, bachelor, undergrad, grad, graduate, undergraduate, scientist, postgrad, faculty, chancellor, university, college, provost, vice-provost |
| Politicians | campaign, campaigner, republican, democrat, senator, mayor, governor, minister, ministry, counselor, parliament, politician, representative, statesman, stateswoman, congressman, congresswoman, spokesman, spokeswoman, senate, chairwoman, chairman, representing, congressional, commissioner, ambassador, congress, councilmember, assembly member, assemblywoman, assemblyman, assembly, councilman, councilwoman congressional, Whitehouse |
| Print and Electronic Media | writer, author, journalist, logger, columnist, communicator, reporter, producer, storyteller, podcaster, youtuber, news, newsman, newswoman, pressman, presswoman, journal, publish, publisher, publishing, peer-reviewed, reviewed, press, elsevier, wiley, emerald, wolters, reutckwell, mdpi, palgrave, routledge, springer, sage, clarivate, bmc, bmj, plos, ieee, cnn, fox, msnbc, bbc, cbs, nbc, abc, wion, cnbc, broadcasterbcgtn, jazeer |
| Healthcare professional | physician, doctor, nurse, midwife, dentist, pharmacist, microbiologist, virologist, oncologist, hematologist, epidemiologist, physiologist, pathologist, endocrinologist, nephrologist, neurologist, geneticist, geriatrician, surgeon, optometrist, ophthalmologist, therapist, podiatrist, chiropodist, pedorthist, pediatrician, immunologist, anesthesiologist, cardiologist, dermatologist, endocrinologist, gastroenterologist, hematologist, obstetrician, gynecologists, osteopath, otolaryngologist, physiatrist, podiatrist, psychiatrist, pulmonologist, radiologist, |
| Healthcare professionals | rheumatologist, urologist, infectious disease, physician, 'infectious disease specialist, 'Johnson & Johnson', renvartis, merck, glaxosmithksanofi, abbvie, takeda, beristol-myers, pharmaceutca, amgen, gilead, biogen, moderna, sinovac, novavax, cfra, allergan, abbott, stryker, regeneron |
| Legal professionals | lawyer, attorney, court, justice, lawmaker, judge, solicitor, barrister, jurist, jury, paralegal, courtroom, arbitrator, legislator |
| Private sector (except drug and pharma companies) | corporation, Inc., ceo, corporate, company, enterprise, cfo, cio, coo, cmo, cfo, cto, cco, firm |

A1. The keywords and hashtags used to find the WHO tweets related to COVID-19

#stayhome, distancing, lockdown, quarantine, isolate, china, wuhan, handwashing, #handwashing, #handhygiene, #safehands, #2019ncov, outbreak, pandemic, masks, vaccines, #vaccines, #coronavirusDrTedros, #UnitedAgainstCoronavirus, #knowTheFact, #KnowTheFacts, #HandHygiene, #COVID19DrTedros, #togetherathome, #covidー19, #coronalockdown, #covidlockdown, #covid19lockdown, #covidquarantine, #coronaquarantine, #coronavirusquarantine, #pandemic, pandemic, #covid-19, #covid19, covid19, #covid, covid, #corona, corona, #coronavirus, coronavirus, #wuhanvirus, #2019novelcoronavirus, #chinacoronavirus, #coronaoutbreak, #coronaphobia, #coronavirusoutbreak, #novelcoronaviruspneumonia, #wuhanconoravirus, #wuhanpneumonia, #coronaviruspandemic, #coronapandemic, #coronacrisis, #coronaviruscrisis, covid2020, #kungflu, #covidpandmeic, #coronapademic, #coronavirusvaccine, #coronavirusvaccines, #coronavirusupdates, #cornaupdates, #covidvaccine, #coronaepidemic, #coronavaccination, #coronavaccine, #postcovid, #covidinfection, #coviddisease, #2019_ncov, #coviddebts, #covidrehab, #SARSCoV2, #covidpositive, #covidrecovery, #covid19Walkout, #coronavirus19, #covidplague, #coronaplague, #covidpandemic, #covidinformation, #covid19plague, #covid19pandemic, #covid19research, #covidresearch, #coronaresearch, #highriskcovid19, #covidhasnoborders, #COVIDー19, #covidsafe, #covidheros

A2. The keywords and hashtags used to find tweets relevant to COVID-19 in the six Twitter user categories

#covidー19, #coronalockdown, #covidlockdown, #covid19lockdown, #covidquarantine, #coronaquarantine, #coronavirusquarantine, #pandemic, pandemic, #covid-19, #covid19, covid19, #covid, covid, #corona, corona, #coronavirus, coronavirus, #wuhanvirus, #2019novelcoronavirus, #chinacoronavirus, #coronaoutbreak, #coronaphobia, #coronavirusoutbreak, #novelcoronaviruspneumonia, #wuhanconoravirus, #wuhanpneumonia, #coronaviruspandemic, #coronapandemic, #coronacrisis, #coronaviruscrisis, covid2020, #kungflu, #covidpandmeic, #coronapademic, #coronavirusvaccine, #coronavirusvaccines, #coronavirusupdates, #cornaupdates, #covidvaccine, #coronaepidemic, #coronavaccination, #coronavaccine, #postcovid, #covidinfection, #coviddisease, #2019_ncov, #coviddebts, #covidrehab, #SARSCoV2, #covidpositive, #covidrecovery, #covid19Walkout, #coronavirus19, #covidplague, #coronaplague, #covidpandemic, #covidinformation, #covid19plague, #covid19pandemic, #covid19research, #covidresearch, #coronaresearch, #highriskcovid19, #covidhasnoborders, #COVIDー19, #covidsafe, #covidheros.

Table S13. Frequency of issues in the datasets of WHO, WHO followers, and each Twitter user category.

|  | WHO | WHOF | H | A | P | PEM | L | PS |
| --- | --- | --- | --- | --- | --- | --- | --- | --- |
| P | 2430 | 228700 | 62414 | 72318 | 17519 | 46921 | 12905 | 16623 |
| So | 717 | 72192 | 18269 | 23500 | 6913 | 12758 | 4286 | 6466 |
| T | 601 | 23879 | 5403 | 7802 | 3267 | 3785 | 1518 | 2104 |
| Su | 276 | 13761 | 4110 | 4443 | 929 | 2419 | 725 | 1135 |
| Ch | 243 | 28870 | 5522 | 9417 | 3131 | 5783 | 2046 | 2971 |
| ill | 213 | 15151 | 3348 | 5160 | 1400 | 2794 | 1282 | 1167 |
| Cr | 156 | 11726 | 3142 | 3766 | 835 | 2567 | 718 | 698 |

WHO Followers (WHOF) which constitutes all six Twitter user categories following WHO on Twitter. H = Healthcare professionals, A = Academics, P = Politicians, PEM = Print and electronic media, L = Legal professionals, PS = Private sector.

P = Prevention, So = Solidarity, T = Teamwork, Su = Surveillance, Ch= Charity, Ill-effect = Ill, Cr = Credibility.

Table S14. The matrix of agenda network for private sector on Twitter.

|  | Teamwork | Charity | Surveillance | Prevention | Solidarity | Ill-effect | Credibility |
| --- | --- | --- | --- | --- | --- | --- | --- |
| Teamwork | 0 | 131 | 27 | 300 | 239 | 27 | 17 |
| Charity | 131 | 0 | 13 | 443 | 563 | 25 | 2 |
| Surveillance | 27 | 13 | 0 | 234 | 158 | 6 | 3 |
| Prevention | 300 | 443 | 234 | 0 | 1234 | 170 | 115 |
| Solidarity | 239 | 563 | 158 | 1234 | 0 | 150 | 49 |
| Ill-effect | 27 | 25 | 6 | 170 | 150 | 0 | 4 |
| Credibility | 17 | 2 | 3 | 115 | 49 | 4 | 0 |

Table S15. The matrix of agenda network for electronic and print media on Twitter.

|  | Teamwork | Charity | Surveillance | Prevention | Solidarity | Ill-effect | Credibility |
| --- | --- | --- | --- | --- | --- | --- | --- |
| Teamwork | 0 | 233 | 49 | 510 | 396 | 40 | 58 |
| Charity | 233 | 0 | 30 | 857 | 996 | 69 | 25 |
| Surveillance | 49 | 30 | 0 | 510 | 136 | 15 | 24 |
| Prevention | 510 | 857 | 510 | 0 | 2476 | 526 | 391 |
| Solidarity | 396 | 996 | 136 | 2476 | 0 | 265 | 159 |
| Ill-effect | 40 | 69 | 15 | 526 | 265 | 0 | 21 |
| Credibility | 58 | 25 | 24 | 391 | 159 | 21 | 0 |

Table S16. The matrix of agenda network for legal professionals on Twitter.

|  | Teamwork | Charity | Surveillance | Prevention | Solidarity | Ill-effect | Credibility |
| --- | --- | --- | --- | --- | --- | --- | --- |
| Teamwork | 0 | 99 | 20 | 220 | 146 | 33 | 17 |
| Charity | 99 | 0 | 11 | 285 | 390 | 34 | 9 |
| Surveillance | 20 | 11 | 0 | 167 | 37 | 7 | 7 |
| Prevention | 220 | 285 | 167 | 0 | 771 | 245 | 132 |
| Solidarity | 146 | 390 | 37 | 771 | 0 | 120 | 50 |
| Ill-effect | 33 | 34 | 7 | 245 | 120 | 0 | 7 |
| Credibility | 17 | 9 | 7 | 132 | 50 | 7 | 0 |

Table S17. The matrix of agenda network for healthcare professionals on Twitter.

|  | Teamwork | Charity | Surveillance | Prevention | Solidarity | Ill-effect | Credibility |
| --- | --- | --- | --- | --- | --- | --- | --- |
| Teamwork | 0 | 226 | 100 | 724 | 533 | 64 | 36 |
| Charity | 226 | 0 | 28 | 868 | 918 | 63 | 18 |
| Surveillance | 100 | 28 | 0 | 943 | 238 | 27 | 30 |
| Prevention | 724 | 868 | 943 | 0 | 3657 | 574 | 688 |
| Solidarity | 533 | 918 | 238 | 3657 | 0 | 319 | 163 |
| Ill-effect | 64 | 63 | 27 | 574 | 319 | 0 | 14 |
| Credibility | 36 | 18 | 30 | 688 | 163 | 14 | 0 |

Table S18. The matrix of agenda network for academics on Twitter.

|  | Teamwork | Charity | Surveillance | Prevention | Solidarity | Ill-effect | Credibility |
| --- | --- | --- | --- | --- | --- | --- | --- |
| Teamwork | 0 | 443 | 114 | 1015 | 759 | 96 | 70 |
| Charity | 443 | 0 | 53 | 1372 | 1625 | 123 | 29 |
| Surveillance | 114 | 53 | 0 | 939 | 275 | 31 | 19 |
| Prevention | 1015 | 1372 | 939 | 0 | 4401 | 841 | 782 |
| Solidarity | 759 | 1625 | 275 | 4401 | 0 | 514 | 227 |
| Ill-effect | 96 | 123 | 31 | 841 | 514 | 0 | 36 |
| Credibility | 70 | 29 | 19 | 782 | 227 | 36 | 0 |

Table S19. The matrix of agenda network for politicians on Twitter.

|  | Teamwork | Charity | Surveillance | Prevention | Solidarity | Ill-effect | Credibility |
| --- | --- | --- | --- | --- | --- | --- | --- |
| Teamwork | 0 | 218 | 39 | 445 | 455 | 62 | 17 |
| Charity | 218 | 0 | 14 | 543 | 589 | 35 | 10 |
| Surveillance | 39 | 14 | 0 | 233 | 64 | 5 | 3 |
| Prevention | 445 | 543 | 233 | 0 | 1305 | 283 | 140 |
| Solidarity | 455 | 589 | 64 | 1305 | 0 | 157 | 70 |
| Ill-effect | 62 | 35 | 5 | 283 | 157 | 0 | 3 |
| Credibility | 17 | 10 | 3 | 140 | 70 | 3 | 0 |

**Granger causality tests results**

Table S20. Granger test of causality of WHO on healthcare professionals and vice versa.

|  | Agendas | F-Test | P-value |
| --- | --- | --- | --- |
| WHO Granger causes  Healthcare professionals | Teamwork | 0.34 | 0.88 |
|  | Charity | 0.39 | 0.86 |
|  | Surveillance | 2.35 | 0.12 |
|  | Prevention | 0.33 | 0.89 |
|  | Solidarity | 0.50 | 0.77 |
|  | Ill-effect | 0.81 | 0.58 |
|  | Credibility | 0.61 | 0.70 |
| Healthcare professionals  Granger causes WHO | Teamwork | 0.19 | 0.96 |
|  | Charity | 37.93 | **0.001** |
|  | Surveillance | 2.03 | 0.16 |
|  | Prevention | 0.78 | 0.58 |
|  | Solidarity | 0.99 | 0.47 |
|  | Ill-effect | 0.85 | 0.55 |
|  | Credibility | 0.60 | 0.71 |

Table S21. Granger test of causality of WHO on academics and vice versa.

|  | Agendas | F-Test | P-value |
| --- | --- | --- | --- |
| WHO Granger causes  Academics | Teamwork | 0.06 | 0.99 |
|  | Charity | 0.32 | 0.91 |
|  | Surveillance | 2.76 | 0.08 |
|  | Prevention | 0.24 | 0.94 |
|  | Solidarity | 0.30 | 0.90 |
|  | Ill-effect | 0.49 | 0.78 |
|  | Credibility | 12.15 | **0.001** |
| Academics Granger  causes WHO | Teamwork | 0.62 | 0.69 |
|  | Charity | 4.62 | **0.04** |
|  | Surveillance | 3.17 | 0.06 |
|  | Prevention | 3.07 | 0.06 |
|  | Solidarity | 0.71 | 0.61 |
|  | Ill-effect | 0.71 | 0.63 |
|  | Credibility | 6.10 | **0.001** |

Table S22. Granger test of causality of WHO on legal professionals and vice versa.

|  | Agendas | F-Test | P-value |
| --- | --- | --- | --- |
| WHO Granger causes  Legal professionals | Teamwork | 0.15 | 0.98 |
|  | Charity | 0.55 | 0.73 |
|  | Surveillance | 3.25 | 0.05 |
|  | Prevention | 0.39 | 0.84 |
|  | Solidarity | 1.25 | 0.37 |
|  | Ill-effect | 1.09 | 0.42* |
|  | Credibility | 0.71 | 0.64 |
| Legal professionals  Granger causes WHO | Teamwork | 2.23 | 0.13 |
|  | Charity | 2.50 | 0.10 |
|  | Surveillance | 3.13 | 0.06 |
|  | Prevention | 1.08 | 0.42 |
|  | Solidarity | 1.76 | 0.23 |
|  | Ill-effect | 0.52 | 0.76* |
|  | Credibility | 1.81 | 0.22 |

* BoxCox transformation was used for this variable

Table S23. Granger test of causality of WHO on politicians and vice versa.

|  | Agendas | F-Test | P-value |
| --- | --- | --- | --- |
| WHO Granger  causes Politicians | Teamwork | 0.19 | 0.96 |
|  | Charity | 0.41 | 0.83 |
|  | Surveillance | 5.13 | **0.03** |
|  | Prevention | 0.41 | 0.83 |
|  | Solidarity | 0.27 | 0.92 |
|  | Ill-effect | 0.86 | 0.54 |
|  | Credibility | 2.80 | 0.09 |
| Politicians Granger  causes WHO | Teamwork | 1.09 | 0.42 |
|  | Charity | 0.63 | 0.68 |
|  | Surveillance | 0.60 | 0.72 |
|  | Prevention | 13.21 | **0.001** |
|  | Solidarity | 0.32 | 0.89 |
|  | Ill-effect | 1.64 | 0.24 |
|  | Credibility | 8.06 | **0.001** |

Table S24. Granger test of causality of WHO on private sector and vice versa.

|  | Agendas | F-Test | P-value |
| --- | --- | --- | --- |
| WHO Granger causes  Private sector | Teamwork | 1.99 | 0.16 |
|  | Charity | 0.04 | 0.99 |
|  | Surveillance | 2.59 | 0.094* |
|  | Prevention | 0.17 | 0.97 |
|  | Solidarity | 1.07 | 0.41 |
|  |  |  |  |
|  | Ill-effect | 1.37 | 0.31 |
|  | Credibility | 7.12 | **0.001** |
| Private sector Granger  causes WHO | Teamwork | 1.07 | 0.39 |
|  | Charity | 2.11 | 0.15 |
|  | Surveillance | 1.48 | 0.28* |
|  | Prevention | 1.05 | 0.45 |
|  | Solidarity | 2.25 | 0.12 |
|  | Ill-effect | 7.35 | **0.001** |
|  | Credibility | 4.33 | **0.02** |

* BoxCox transformation was used for this variable

Table S25. Granger test of causality of WHO on print and electronic media and vice versa.

|  | Agendas | F-Test | P-value |
| --- | --- | --- | --- |
| WHO Granger  causes Media | Teamwork | 0.06 | 0.99 |
|  | Charity | 0.32 | 0.90 |
|  | Surveillance | 9.33 | **0.001** |
|  | Prevention | 0.21 | 0.94 |
|  | Solidarity | 0.37 | 0.86 |
|  | Ill-effect | 4.02 | **0.02** |
|  | Credibility | 0.52 | 0.72 |
| Media Granger  causes WHO | Teamwork | 0.57 | 0.71 |
|  | Charity | 4.25 | .051 |
|  | Surveillance | 3.7 | **0.04** |
|  | Prevention | 15.04 | **0.001** |
|  | Solidarity | 0.49 | 0.78 |
|  | Ill-effect | 0.52 | 0.72 |
|  | Credibility | 2.5 | 0.1 |
