# Supplementary figures and images for: The Mutual Influence of the World Health Organization (WHO) and Twitter Users During COVID-19: Network Agenda-Setting Analysis
Source: J Med Internet Res. 2022 Apr 26;24(4):e34321. doi: 10.2196/34321 (PMC9045487; doi:10.2196/34321)

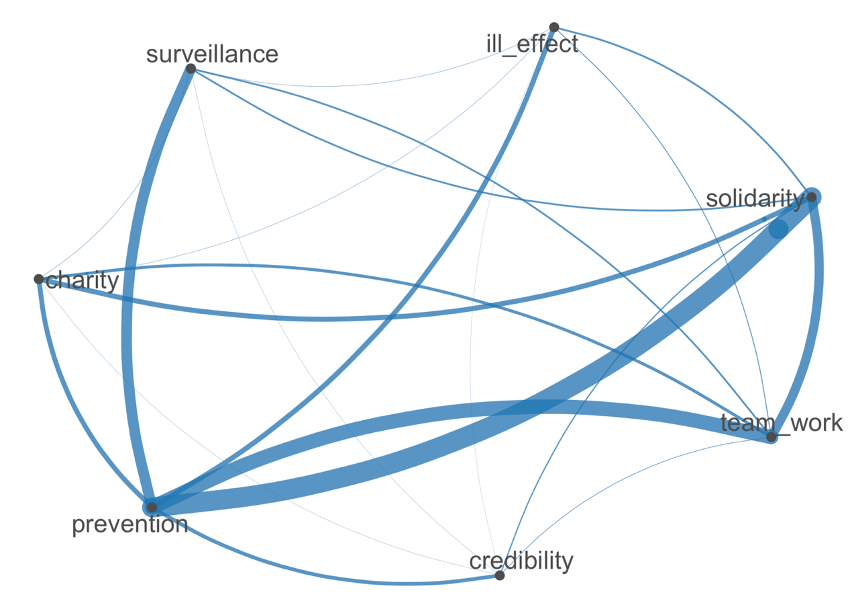

Supplement: Multimedia Appendix 2 [file jmir_v24i4e34321_app2.png]

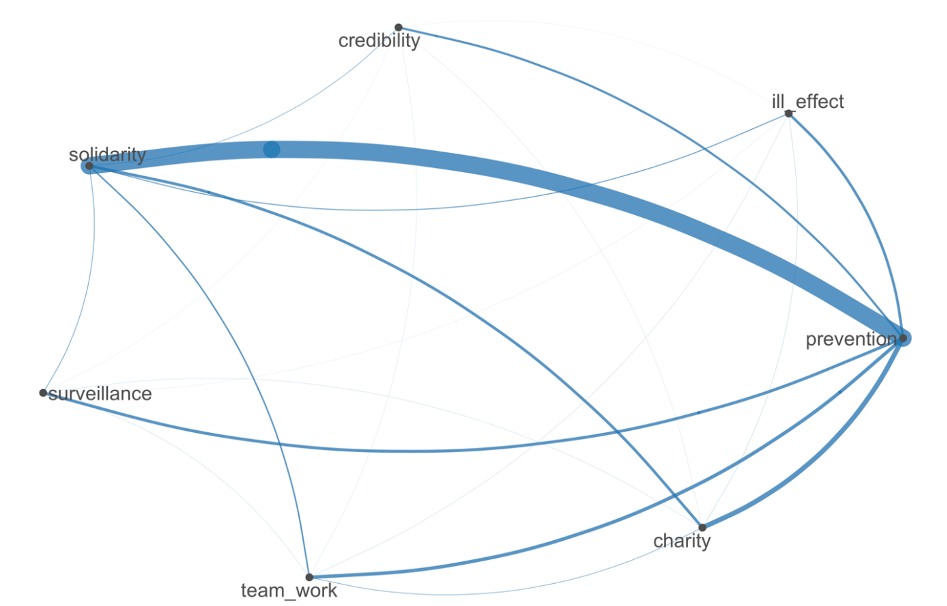

Supplement: Multimedia Appendix 3 [file jmir_v24i4e34321_app3.png]

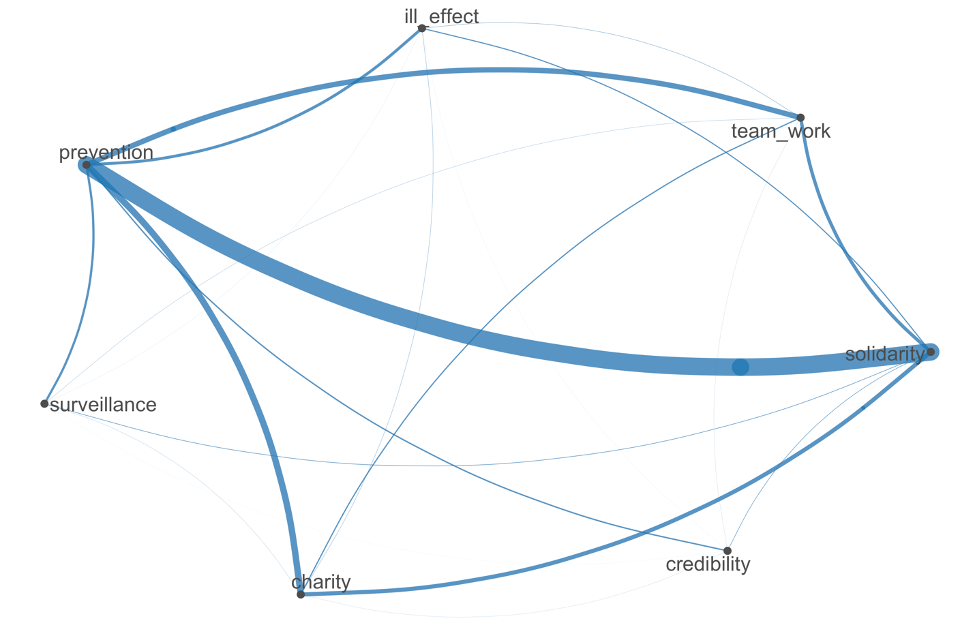

Supplement: Multimedia Appendix 4 [file jmir_v24i4e34321_app4.png]

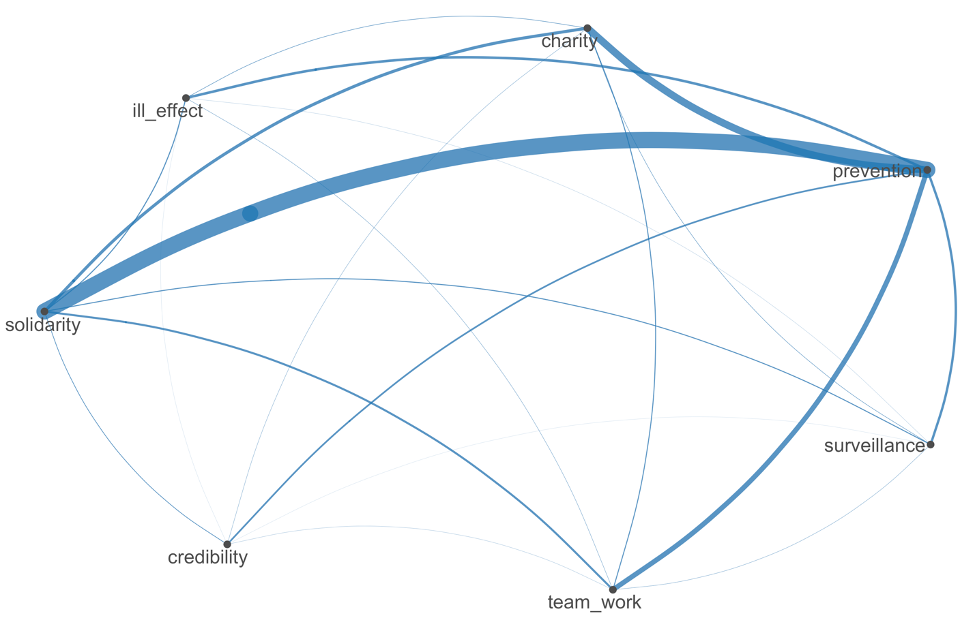

Supplement: Multimedia Appendix 5 [file jmir_v24i4e34321_app5.png]

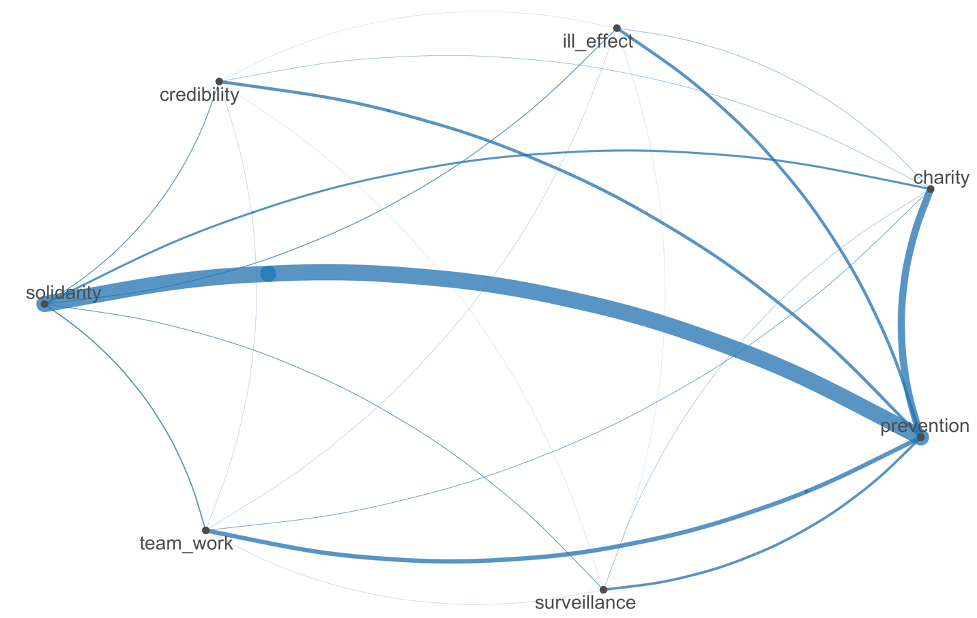

Supplement: Multimedia Appendix 6 [file jmir_v24i4e34321_app6.png]

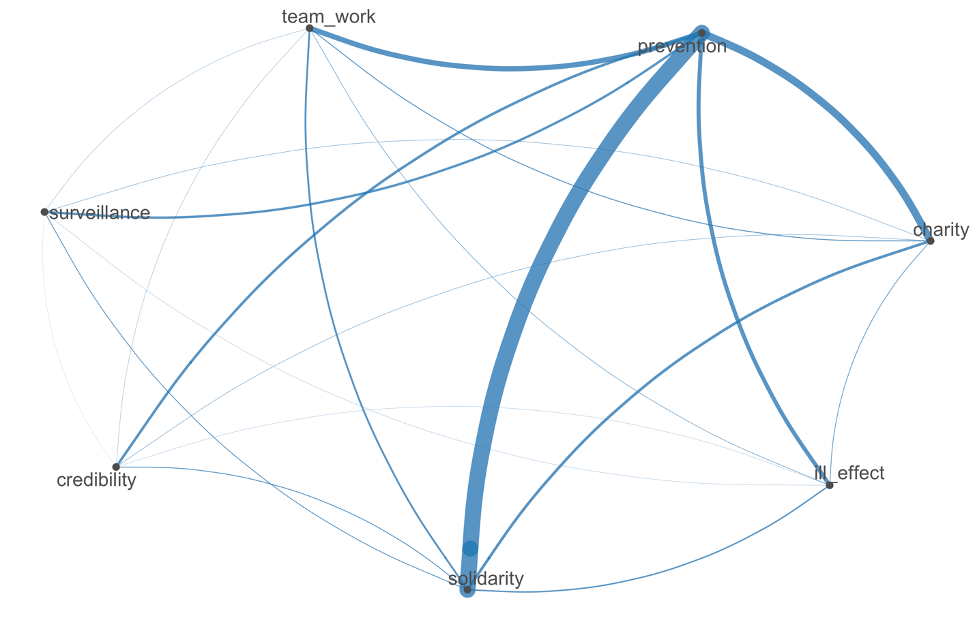

Supplement: Multimedia Appendix 7 [file jmir_v24i4e34321_app7.png]

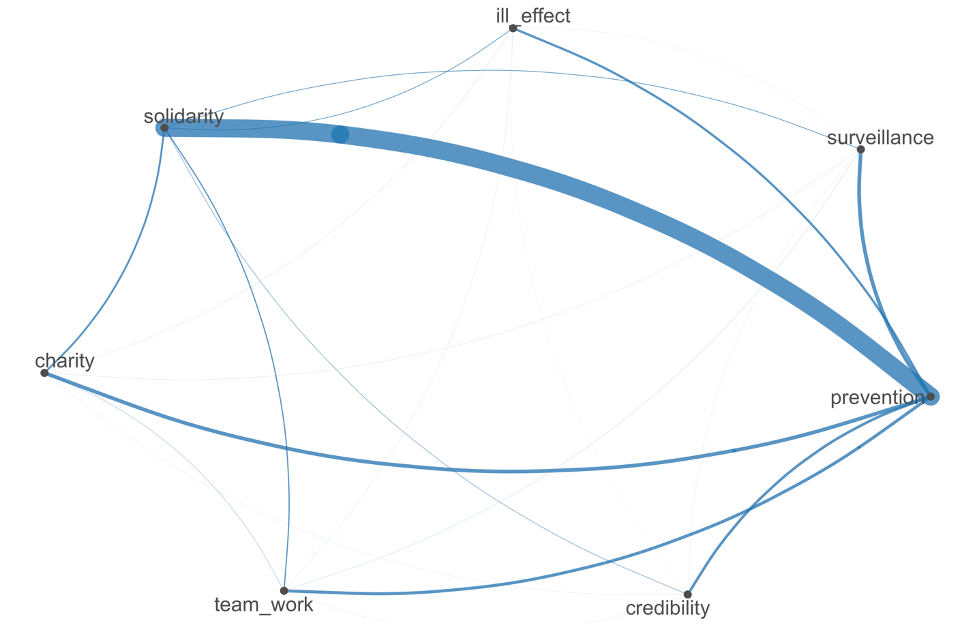

Supplement: Multimedia Appendix 8 [file jmir_v24i4e34321_app8.png]
